# A Reexamination of the Carnivora Malleus (Mammalia, Placentalia)

**DOI:** 10.1371/journal.pone.0050485

**Published:** 2012-11-27

**Authors:** John R. Wible, Michelle Spaulding

**Affiliations:** Section of Mammals, Carnegie Museum of Natural History, Pittsburgh, Pennsylvania, United States of America; Monash University, Australia

## Abstract

Authoritative anatomical references depict domestic dogs and cats as having a malleus with a short rostral (anterior) process that is connected via a ligament to the ectotympanic of the auditory bulla. Similar mallei have been reported for representatives of each of the 15 extant families of Carnivora, the placental order containing dogs and cats. This morphology is in contrast to a malleus with a long rostral process anchored to the ectotympanic that is considered to be primitive for mammals. Our reexamination of extant carnivorans found representatives from 12 families that possess an elongate rostral process anchored to the ectotympanic. Consequently, the malleus also is a component of the bulla. In a subset of our carnivoran sample, we confirmed that the elongate rostral process on the ectotympanic is continuous with the rest of the malleus through a thin osseous lamina. This morphology is reconstructed as primitive for Carnivora. Prior inaccurate descriptions of the taxa in our sample having mallei continuous with the bulla were based on damaged mallei. In addition to coupling to the ectotympanic, the rostral process of the malleus was found to have a hook-like process that fits in a facet on the skull base in representatives from seven families (felids, nandiniids, viverrids, canids, ursids, procyonids, and mustelids); its occurrence in the remaining families could not be ascertained. This feature is named herein the mallear hook and is likewise reconstructed to be primitive for Carnivora. We also investigated mallei in one additional placental order reported to have mallei not connected to the ectotympanic, Pholidota (pangolins), the extant sister group of Carnivora. We found pholidotans to also have anchored mallei with long rostral processes, but lacking mallear hooks. In light of our results, other mammals previously reported to have short rostral processes should be reexamined.

## Introduction

One key distinguishing feature of extant mammals is a chain of three middle-ear ossicles: the malleus, incus, and stapes, which transmit sound from the outer ear and tympanic membrane to the inner ear. Over the last few years, incredibly well-preserved fossils of Mesozoic mammals and near relatives have greatly improved our understanding of the transformation of these bones from primary load-bearing elements of the jaw joint in non-mammalian cynodonts (the articular and quadrate) to the suspended malleus and incus of mammals [1], [2], [3], [4]. Even though the suspension of the three ossicles may have occurred convergently in monotremes and in therians (marsupials and placentals), as suggested by some [3], [4], [5], the presence of the malleus, incus, and stapes remains one of the few and most recognizable osseous hallmarks of mammals.

Despite the recent increased understanding of the Mesozoic origins of the three auditory ossicles, our knowledge of this complex across the diversity of extant mammals still has significant gaps. Even today, all that is known for several groups can be traced to the famous 1878 monograph by Alban Doran [6], who described and illustrated these bones in multiple representatives of all extant orders. There are practical reasons for the gaps: these bones are usually tiny, fragile, and cryptic, hidden by the bony auditory bulla that encloses the middle ear in many therians. High-resolution computed tomography (CT) is helping to address these obstacles, resulting in significant contributions for some groups (e.g., [7], [8], [9]).

The comparative anatomy of the ossicles of the placental order Carnivora (cats, dogs, bears, civets, and relatives) has received more attention than those of most clades. This is both because the systematic value of the ossicles, in particular the malleus, long has been recognized [10], [11], [12], [13] and because the domestic cat and dog, *Felis catus* and *Canis lupus familiaris*, have been the subjects of numerous detailed anatomical treatises [14], [15], [16], [17]. These two species are second only to humans in their level of prior anatomical study and are commonly used as reference points in comparative anatomical studies even when carnivorans are not the focal point of the research.

**Figure 1:**
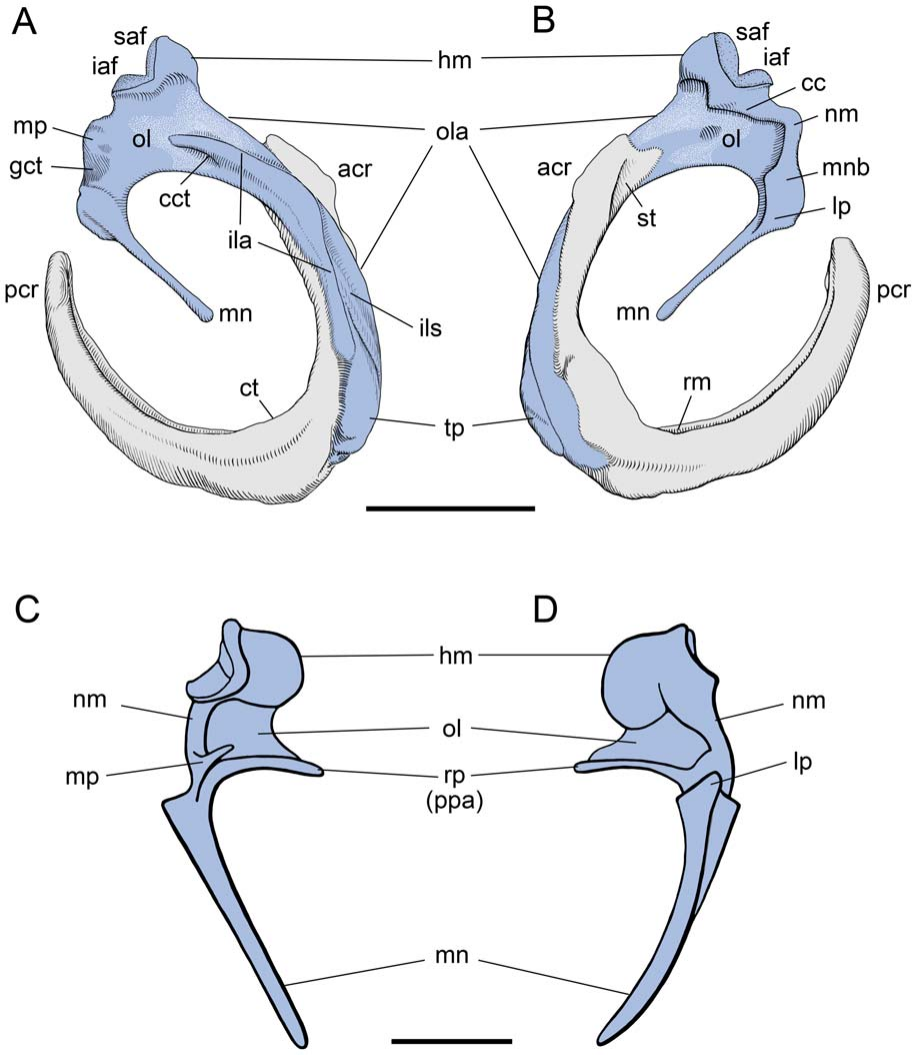
Left ectotympanic (gray) and malleus (blue) in medial (A, C) and lateral (B, D) views. **A, B,** *Didelphis virginiana*, CM 39794 (reversed from right specimen). **C, D,** *Felis catus*, (redrawn from figs. 152, 154 in [16]), showing the widely accepted morphology and terminology of the carnivoran malleus. Scale bars  = 2 mm. Abbreviations: **acr,** anterior crus of ectotympanic; **cc,** capitular crest; **cct,** canal for chorda tympani; **ct,** crista tympanica; **gct,** groove for chorda tympani; **hm,** head of malleus; **iaf,** inferior articular surface; **ila,** inner lamella; **ils,** interlamellar sulcus; **lp,** lateral process; **mnb,** manubrial base; **mn,** manubrium; **mp,** muscular process; **nm,** neck of malleus; **ol,** osseous lamina; **ola,** outer lamella; **pcr,** posterior crus of ectotympanic; **ppa,** pars processus anterioris; **rm,** recessus meatus; **rp,** rostral process; **saf,** superior articular facet; **st,** sulcus tympanicus; **tp,** tympanic plate of rostral process.

To date, mallei from nearly 100 carnivoran species, including representatives from each of the 15 currently recognized families [18], have been studied [6], [10], [11], [12], [13], [19], [20], [21]. Mallei with the primitive condition of basal therians, as exemplified by the Virginia opossum, *Didelphis virginiana* [4], [22], are tightly coupled or anchored via an elongate rostral (anterior) process to the ectotympanic bone, from which the tympanic membrane is suspended (Fig. 1A, B). In contrast, the carnivoran malleus, as exemplified by the domestic cat (Fig. 1C, D), has been reported to have at best a rudimentary rostral process that is not tightly coupled to the ectotympanic. This type of malleus has been presented as a near universal pattern for carnivorans [12].

Nevertheless, discrepancies in the literature exist in the reports of some carnivoran taxa. The widely described carnivoran pattern, with small rostral process (Fig. 1C, D), has been attributed by most anatomists to the canids *Canis* [6], [12], [15], [17], [20] and *Vulpes* [6], the otariid *Arctocephalus pusillus* [6], the phocid *Phoca vitulina* [6], [20], and the mustelid *Martes* [6], [10]. In contrast, for these same forms, Fleischer [21] reported a long rostral process anchored to the ectotympanic, resembling the primitive condition as exemplified by *D. virginiana* (Fig. 1A, B), and included a photograph of that condition in a newborn wolf. Fleischer [21] did not comment on this discrepancy; nor have subsequent authors. Additionally, in *Felis catus*, the short rostral process is reported to have a ligamentous connection to the middle-ear wall in the anatomical literature (e.g., [16]), but is said to be fused in the physiological acoustics literature [23]. To assess these discrepancies, we present here a reexamination of the carnivoran malleus.

## Materials and Methods

The osteological collection of the Section of Mammals, Carnegie Museum of Natural History (CM), Pittsburgh, PA, was the major source for specimens, supplemented from the Department of Mammalogy, American Museum of Natural History (AMNH), New York, NY. Our study sample consisted not only of adults, which usually had fused sutures between at least some bones surrounding the middle ear, but included newborns and juveniles when available. Mallei were not deliberately removed from skulls for this study, because of concerns of breakage, but studied in situ and as isolated elements discovered in the bottom of specimen boxes. The bony auditory bulla was the major impediment to studying the malleus in situ, so we searched for specimens with open bullae or opened the bullae ourselves. As reports on mammalian auditory ossicles generally do not detail the methods by which these fragile structures were obtained, we do not know how our methods compare to those of prior studies.

A standard terminology of the auditory ossicles and middle ear does not exist. Many researchers use the Nomina Anatomica Veterinaria [24], abbreviated NAV here, when identifying structures. However, NAV only includes six terms for structures of the malleus, which is inadequate to describe the diversity of this complex element across mammals. Therefore, we supplement the limited NAV terminology with others in the literature, primarily relying on Henson [25]. Our usages and synonyms for terms of the malleus and ectotympanic are included in Table 1; sources for other terms can be found in Wible [26], [27]. Of particular importance for this report is what constitutes a rostral process (anterior process of human anatomy [28]). We follow Henson [25] in considering the rostral process as composed of three principal parts: the osseous lamina, the pars processus anterioris (PPA), and the tympanic plate.

**Table 1:** Anatomical terms for the malleus and ectotympanic employed here with sources and synonyms.

Terms Employed in This Study	Sources and Synonyms
Anterior Crus of Ectotympanic	[26], [27]; Annulus Tympanicus, Crus Anterior [24]; Anterior Leg of Tympanic [25]
Base of Tympanic Plate of Rostral Process	[This study]
Capitular Crest of Malleus	[25]
Capitular Spine of Malleus	[25]
Central Buttress of Malleus	[25]
Crista Tympanica	[25]
Ectotympanic	[26], [27]; Os Temporale, Pars Tympanica [24]; Tympanic [25]
Ectotympanic Notch	[This study]
Foramen for Chorda Tympani of Malleus	[25]
Gonial	[32]; Goniale [22]
Head of Malleus	[25]; Caput Mallei [24]
Inferior Articular Facet of Malleus	[25]
Inner Lamella	[25]
Interlamellar Sulcus	[25]
Lateral Ligament of Malleus	[25]
Lateral Process of Malleus	[25]; Malleus, Processes Lateralis [24]; Short Process [16]
Mallear Hook	[This study]; Petrosal Process of Rostral Process [26]; Ectotympanic Hook [16]
Malleus	[24]
Manubrial Base	[25]
Manubrium of Malleus	[25]; Manubrium Mallei [24]; Handle [16]
Meckel’s Cartilage	[32]
Membrane Margin of Manubrium	[25]
Muscular Process of Malleus	[25]; Malleus, Processus Muscularis [24]; Process for Tensor Tympani [16]
Neck of Malleus	[25]; Collum Mallei [24]
Osseous Lamina of Malleus	[17]; Lamina [16], [25]; Anterior Lamina [12]; Transversal Lamina [57]
Outer Lamella	[25]
Pars Processus Anterioris	[25]
Posterior Crus of Ectotympanic	[26], [27]; Annulus Tympanicus, Crus Posterior [24]; Posterior Leg of Tympanic [25]
Recessus Meatus	[44]; Recessus Meatus Acousticus Externi [25]
Rostral Ligament of Malleus	[17]
Rostral Process of Malleus	[26], [27]; Malleus, Processus Rostralis [24]; Long Process [16]; Folian Process [44]; Processus Gracilis [8]; Anterior Process [12], [22], [28]; Gonial Process [4]; Goniale [21]
Spine of Tympanic Plate of Rostral Process	[This study]
Sulcus Malleolaris	[44]
Sulcus Tympanicus	[24]
Superior Articular Facet of Malleus	[25]
Tympanic Incisure	[26], [27]; Tympanic Notch, Incisura Tympanica, Incisura of Rivinus [25]
Tympanic Margin of Manubrium	[25]
Tympanic Plate of Malleus	[25]

To facilitate comparisons, we illustrate all specimens as left sides, noting when elements have been reversed to conform.

## Background

### *Didelphis virginiana*

The three middle-ear ossicles and ectotympanic of the Virginia opossum have been illustrated often as exemplars in contributions on the evolution of the mammalian auditory apparatus (e.g., [4], [22]). Despite this, the opossum malleus and ectotympanic have not heretofore been adequately described in full; consequently, our descriptions begin with this taxon.

The opossum ectotympanic lies in a near vertical plane posteromedial to the jaw joint. The malleus is coupled to the ectotympanic bone (Fig. 1A, B), and the two bones lack any direct bony contact to the skull base. Instead, the malleus articulates with the incus, which in turn articulates with the stapes. Both the incudomallear and incudostapedial joints are reported to be synovial [29]. The incus and stapes contact the petrosal (petrous temporal) bone, which houses the inner ear: the crus breve of the incus is held by the caudal incudal ligament in a depression, the fossa incudis, and the footplate of the stapes is held by the annular ligament in the fenestra vestibuli (oval window).

The malleus and ectotympanic can be visualized roughly as U-shaped structures that point in opposite directions (Fig. 1A, B). The ectotympanic is a larger, more regular U with anterior and posterior legs or crura of near equal length. The wide gap between the crura, the tympanic incisure, is directed posterodorsally in the intact skull and houses the pars flaccida of the tympanic membrane. The inner surface of the ectotympanic has a well-developed sulcus, the sulcus tympanicus, the medial margin of which is the crista tympanica, which provides attachment for the pars tensa of the tympanic membrane. The ectotympanic is not of uniform girth, but is slightly expanded laterally in its central portion, forming a narrow floor for the recessus meatus, the most proximal part of the external acoustic meatus. The anterior face of the anterior crus has a sulcus malleolaris for the tympanic plate of the rostral process of the malleus (see below).

The malleus (Fig. 1A, B) has a more irregular shape, with a central dorsal portion (including the head) and two anteroventral projections: a larger, longer, curved anterior one (the tympanic plate of the rostral process) lying within the sulcus malleolaris of the anterior crus of the ectotympanic and a shorter, thin, straight, freestanding posterior one (the manubrium). The latter projects slightly medially and does not lie in the same near parasagittal plane as the former. Dorsally on the central portion is the compact head. The posterodorsal aspect of the head is dominated by two articular surfaces set at roughly 90° to each other that contact the incus: a slightly larger, flat superior articular facet and a saddle-shaped inferior one. In lateral view (Fig. 1B), the head is set off by a pronounced capitular crest. This crest has a distinct tubercle resembling that described in some microchiropterans for the attachment of the lateral ligament of the malleus [25]; however, the lateral ligament is lacking in the opossum [29].

In lateral view (Fig. 1B), ventral to the head, the capitular crest continues along the posterodorsal margin, makes a 90° turn, and bulges as the manubrial base. This thick, sigmoidal area between the head and the manubrial base is the neck of the malleus. Extending anteroventrally from the manubrial base is the manubrium, which is strongly anteroposteriorly compressed and shows no distal expansion. The tympanic (medial) margin of the manubrium is flat, but the membrane (lateral) margin tapers towards the distal end, making the manubrium wider proximally than distally. On the lateral side, the juncture of the manubrium and manubrial base is the site of the lateral process, which marks the dorsalmost contact of the tympanic membrane on the manubrium. The lateral process is not well differentiated in the opossum. On the medial side (Fig. 1A), dorsal to the manubrial base is a faint, shallow concavity that marks the passage of the chorda tympani nerve, a branch of the facial nerve (cranial nerve VII). Dorsal to that is a faint muscular process for the attachment of the tensor tympani muscle.

Ventral to the head and anterior to the manubrial base is the paper-thin osseous lamina (Fig. 1A, B), which is the proximal part of the rostral process. The distal part of the rostral process is the tympanic plate, which follows the curve of and is affixed to the sulcus malleolaris on the anterior surface of the anterior crus of the ectotympanic. The proximal third of the tympanic plate lies in the same plane as the osseous lamina and is narrow; the distal two-thirds are perpendicular to the proximal part and broader. At its distal terminus, the tympanic plate is tongue-shaped. Arising centrally from and lying perpendicular to the medial surface of the osseous lamina is a well-developed crest, the inner lamella (Fig. 1A), which curves anteroventrally to become the medial margin of the tympanic plate. Proximally, a canal transmitting the chorda tympani pierces the base of the inner lamella. Another crest, the outer lamella, begins anteroventral to the head on the osseous lamina and continues anteroventrally to become the lateral margin of the tympanic plate. In the central third of the tympanic plate, the inner and outer lamellae approximate each other, forming a narrow interlamellar sulcus.

**Figure 2:**
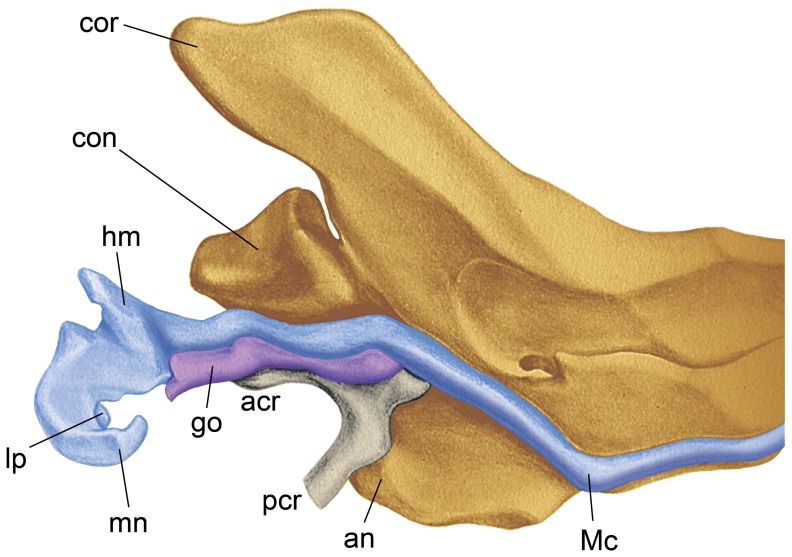
*Didelphis marsupialis*, 45.5 mm long pouch young, medial view of the posterior left lower jaw (yellow), showing the relationships of Meckel’s cartilage and the part of the malleus derived from it (blue), gonial (purple), and ectotympanic (gray); reversed and modified from Taf. III, fig. 9 in [30]). The ventral surface of the posterior aspect of the gonial is pierced by a foramen for the chorda tympani nerve (not visible). Abbreviations: **acr,** anterior crus of ectotympanic; **an,** angular process; **con,** condylar process; **cor,** coronoid process; **go,** gonial; **hm,** head of malleus; **lp,** lateral process; **Mc,** Meckel’s cartilage; **mn,** manubrium; **pcr,** posterior crus of ectotympanic.

The ontogeny of the malleus and ectotympanic in the opossum [29], [30], [31] follows the pattern that has been described widely in other mammals [32]. The central portion (head) and manubrium of the malleus arise from the caudal part of Meckel’s cartilage (Fig. 2), the first branchial arch cartilage. Rostrally, this cartilage provides the scaffold for the intramembranous lower jaw bone (mandible). These parts of the malleus are first distinguished from the remaining rod-shaped Meckel’s cartilage on day twelve of gestation [29], with birth occurring on day thirteen [31]. Two small intramembranous bones, the gutter-like gonial and the Y-shaped ectotympanic (Fig. 2), first appear one and a half days after birth ventral to the caudal part of Meckel’s cartilage [29]. The gonial forms within the perichondrium of Meckel’s cartilage, lies posterior to the ectotympanic, and is pierced by the chorda tympani nerve [29]. In subsequent stages, the gonial expands anteriorly between Meckel’s cartilage and the ectotympanic, and posteriorly to contact the central part of the malleus [29]. At the latter contact, Meckel’s cartilage disintegrates in a craniad direction [29].

Consequently, the adult malleus is a compound structure: part is derived from Meckel’s cartilage and part from the gonial. These elements respectively correspond to the articular and prearticular of non-mammalian cynodonts [4], [22], [33]. The exact position of the line of demarcation between the two parts in the adult opossum is uncertain because the two are fused. On the medial surface (Fig. 1A), it is must be near the origin of the inner lamella, because the chorda tympani passes though the gonial in prenatal and pouch young stages as it does the inner lamella of the adult. At least part of the proximal rostral process (osseous lamina) is likely Meckelian in origin and the distal rostral process (tympanic plate) is gonial. A small piece of cartilage that first appears on postnatal day two in the opossum and is subsequently incorporated into the lateral ridge that connects the mallear head and manubrial base has been hypothesized to be a remnant of the surangular [29], another accessory jaw bone in many non-mammalian cynodonts [34]. However, this is unlikely in light of the position of the surangular dorsal to the prearticular in these forms, which suggests that if present in the opossum it should be near the rostral process (gonial).

It has recently been reported in fate-mapping studies of laboratory mice embryos that the tissues forming the lateral process of the malleus are derived from the second branchial arch [35], which is in contrast to the usual notion of first arch origin. Because the retroarticular process of the articular in the chicken is also of second arch origin [36], [37], it has been suggested that the retroarticular process of non-mammalian cynodonts is equivalent to the lateral process of the mammalian malleus and that the manubrium is a mammalian neomorph [4].

### Carnivora

To date, the most striking difference in the mallei reported for extant members of the placental order Carnivora compared to the mallear bauplan exemplified by *D. virginiana* is in the morphology of the rostral process, specifically the portion designated the tympanic plate. Here, we begin with the anatomy reported for the domestic cat and dog in commonly cited texts.

#### *Felis catus*

**Figure 3:**
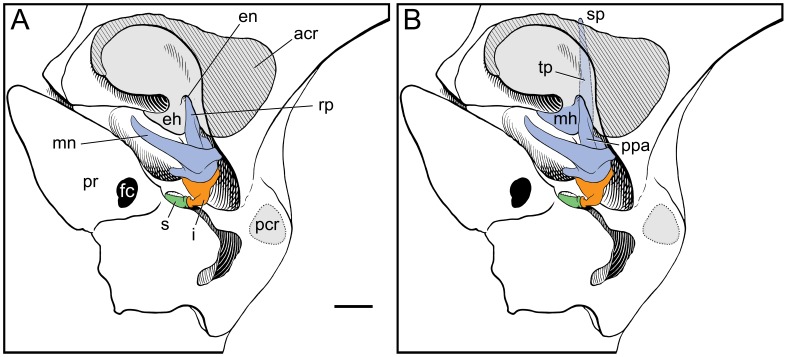
*Felis catus*, adult, ventral views of left auditory region with auditory bulla removed (parallel lines represent cut edge of anterior crus of ectotympanic); modified from fig. 174 in [16]. **A,** Jayne’s [16] identification of the relationship between the rostral process of the malleus (blue) and the ectotympanic (gray). **B,** our interpretation of the relationships between the rostral process of the malleus (blue) and the ectotympanic (gray). Scale bar = 2 mm. Abbreviations: **acr,** anterior crus of ectotympanic; **eh,** ectotympanic hook; **en,** ectotympanic notch; **fc,** fenestra cochleae (round window); **i,** incus; **mh,** mallear hook; **mn,** manubrium; **pcr,** attachment of posterior crus of ectotympanic; **ppa,** pars processus anterioris; **pr,** promontorium of petrosal; **rp,** rostral process; **s,** stapes; **sp,** spine of tympanic plate; **tp,** tympanic plate of rostral process.

The most detailed and best-illustrated description of the adult skull in the cat is Jayne [16]. He figured (figs. 152–155) the malleus in medial, lateral, posterior, and anterior views (the first two views are reproduced here in Fig. 1C, D) and also in situ attached to the ectotympanic (reproduced here in Fig. 3A). The osseous lamina has a thickened ventral margin that is identified as the rostral process, which following Henson [25] is the pars processus anterioris (PPA). The rostral process is very short, barely extending past the anteroventral termination of the osseous lamina (Fig. 1C, D); a tympanic plate is wholly lacking. The thin osseous lamina is reported to often break when the ossicles are removed, but no mention is made of breakage of the rostral process. The pointed end of the rostral process is said to fit in a pit on the inner aspect of the ectotympanic, called the ectotympanic notch here (Fig. 3A), and is held in place there by a ligament; the free margin of the osseous lamina is attached by membrane there as well. Posteromedial to the pit for the rostral process is a claw-shaped process called the hook of the ectotympanic.

As noted above, in the physiological acoustics literature, the cat rostral process is described as “fused to the tympanic wall” (p. 1 in [23]). However, additional details of this fusion have not been reported.

A prior study of the development of the cat skull [38] does not include enough detail on the malleus to make comparisons with the ontogenetic pattern well known for the opossum. A separate gonial is not described. However, given that the tympanic plate is missing, the gonial, if present, must be a minor contributor to the adult malleus.

#### *Canis lupus familiaris*

Evans [17] is the most oft cited current anatomical reference on the domestic dog. The malleus is figured in medial and posterior views, as well as ventrally in situ (see figs. 20–11A, B, 20–12 in [17]). Here, as in the cat, the rostral process is illustrated as barely extending past the edge of the osseous lamina and the tympanic plate is lacking. It is reported that the rostral process is “largely embedded in the tympanic membrane” and attached to the ectotympanic by the rostral ligament of the malleus.

Prior studies of the development of the dog skull [17], [39] identify a separate gonial. It resembles that in the opossum (Fig. 2), except that it is not pierced by the chorda tympani nerve. The fate of the gonial is not reported, but as with the cat, it must be a minor contributor to the adult malleus.

#### Other Carnivora

A similar short rostral process (and even shorter in pinnipeds) is reported and commonly figured in the literature for at least one taxon in every family of carnivorans [6], [10], [11], [12], [13], [19], [20]. As noted above, the only exceptions are found in Fleischer [21] who reported a long rostral process with a tympanic plate coupled to the ectotympanic in two canids, an otariid, a phocid, and a mustelid. Fleischer [see fig. 51 in [21]) included photographs of two newborn wolves, *Canis lupus*, that clearly show the rostral process is long and in articulation with the ectotympanic, just as in *Didelphis* (Fig. 1A, B).

Reports on the development of the skull in carnivorans other than cats and dogs do not add detail about the ontogeny of the malleus (summarized in [32]).

## Results

Our results are arranged by family, beginning with the six families of Feliformia (cat-line carnivorans) and followed by the nine families of Caniformia (dog-line carnivorans).

### Felidae

#### *Panthera pardus*

**Figure 4:**
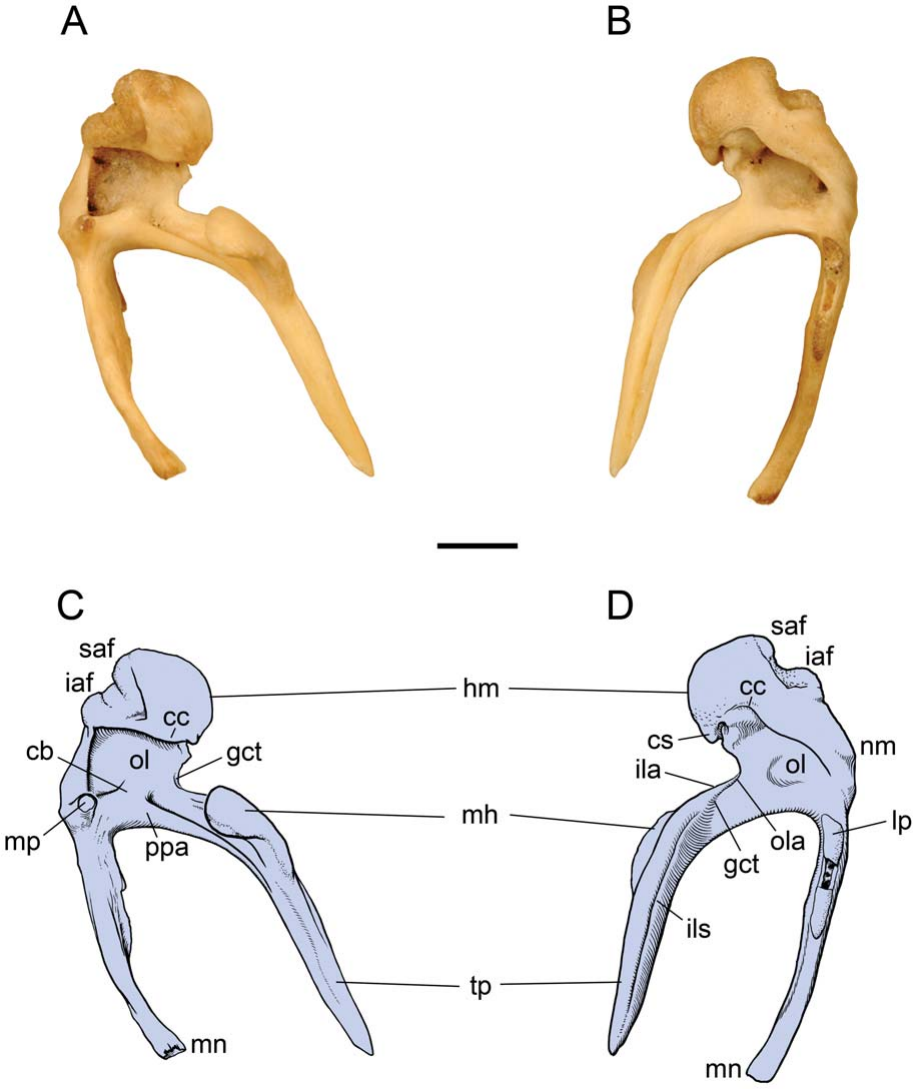
*Panthera pardus*, CM 21066, left malleus (reversed from right specimen). **A, C,** medial; **B, D,** lateral views. Scale bar = 2 mm. Abbreviations: **cb,** central buttress; **cc,** capitular crest; **cs,** capitular spine; **gct,** groove for chorda tympani; **hm,** head of malleus; **iaf,** inferior articular facet; **ila,** inner lamella; **ils,** interlamellar sulcus; **lp,** lateral process; **mh,** mallear hook; **mn,** manubrium; **mp,** muscular process; **nm,** neck of malleus; **ol,** osseous lamina; **ola,** outer lamella; **ppa,** pars processus anterioris; **saf,** superior articular facet; **tp,** tympanic plate of rostral process.

**Figure 5:**
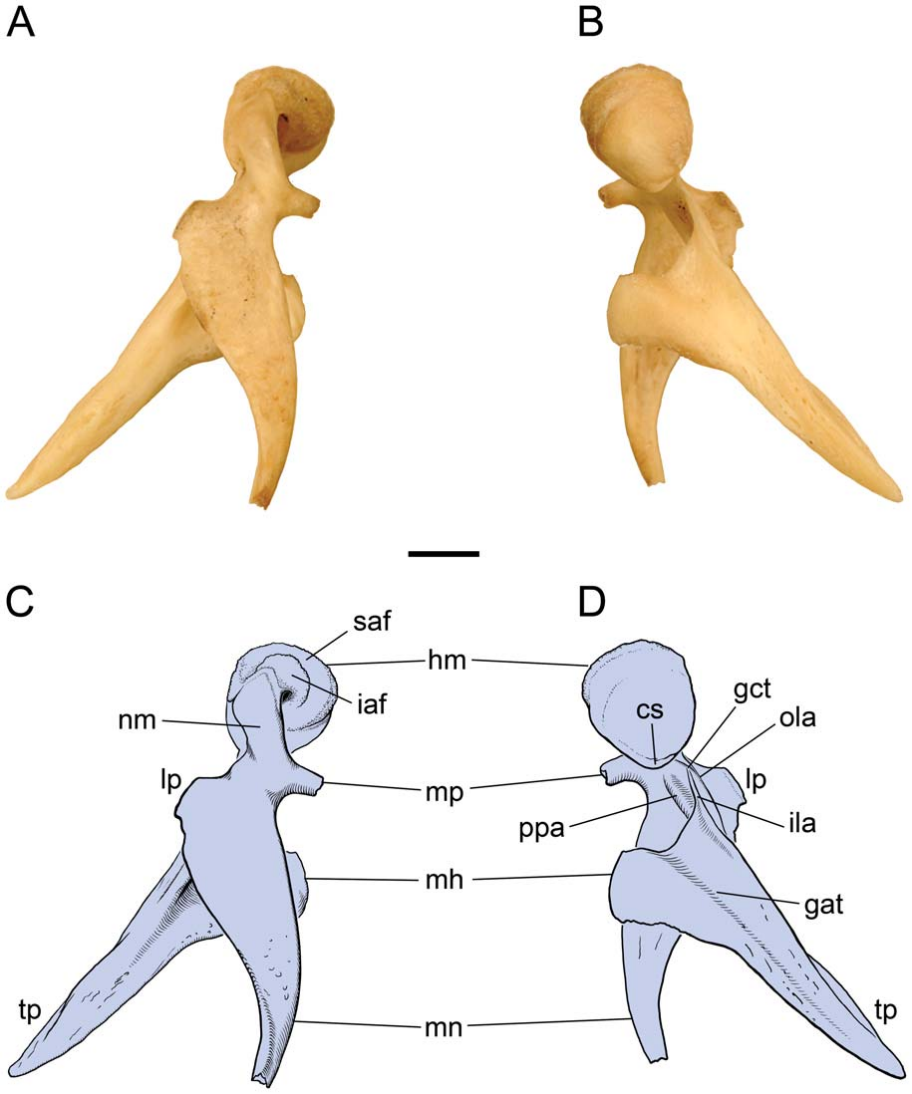
*Panthera pardus*, CM 21066, left malleus (reversed from right specimen). **A, C,** posterior; **B, D,** anterior views. Scale bar = 2 mm. Abbreviations: **cs,** capitular spine; **gat,** groove for anterior tympanic artery; **gct,** groove for chorda tympani; **hm,** head of malleus; **iaf,** inferior articular facet; **ila,** inner lamella; **lp,** lateral process; **mh,** mallear hook; **mn,** manubrium; **mp,** muscular process; **nm,** neck of malleus; **ola,** outer lamella; **saf,** superior articular facet; **tp,** tympanic plate of rostral process.

CM 21066, juvenile male leopard with almost fully erupted deciduous dentition; the ectotympanics are preserved in situ bilaterally; both mallei were found in the bottom of the specimen box (Figs. 4, 5); no incus or stapes is preserved nor are entotympanics, which along with the ectotympanic form the adult auditory bulla.

The central portion of the malleus is dominated by the globose, well-developed head (Figs. 4, 5). The posterior face of the head is the superior articular facet for the incus (Figs. 4A, C, 5A, C). Posterior to that, the inferior articular facet lies at more than a 90° angle to and is about one-half the surface area of the superior one (Figs. 4A, C, 5A, C). Both facets are saddle-shaped and are covered with spongy bone, indicative of their juvenile stage. Anteroventromedially, the head comes to a small, rounded point, a capitular spine (Figs. 4B, D, 5B, D). The head is delimited by a well-developed capitular crest that nearly encircles the base (Fig. 4); only a small area on the anterolateral aspect lacks a well-developed crest (Fig. 4B, D). The crest continues through the neck to the manubrial base on both the medial and lateral surfaces, and is much thicker on the lateral surface.

Unlike the opossum, the neck is not sigmoidal but relatively straight, and the manubrial base is not indicated by a distinct bulge; it is merely the confluence of the neck, manubrium, lateral process, and ventral margin of the osseous lamina (Figs. 4, 5A, C). The manubrium is anteroposteriorly constricted (Fig. 4) and tapers distally (Fig. 5). At its proximal end is the well-developed lateral process (Fig. 5). In lateral view (Fig. 4B, D), the manubrium has two areas of unfinished spongy bone: distally at the tip and proximally at and ventral to the lateral process. On the medial surface, the stout, cylindrical muscular process lies at 90° to the manubrial base (Figs. 4A, C, 5). The course of the chorda tympani nerve is not marked near the muscular process, but it likely passes ventral to it as reported in other felids [40].

The quadrangular osseous lamina is between the head, neck, and manubrial base (Fig. 4). Dorsally, it is composed of thin bone, but ventrally it has a thick margin on both the medial and lateral aspects. This thick margin is the rostral process of Jayne [16], the PPA of Henson [25], which is absent in the opossum (Fig. 1A, B). The PPA is more developed on the medial surface (Fig. 4A, C), where it has a central column extending dorsally a short distance called the central buttress. The PPA and osseous lamina taper anteriorly as they extend onto the proximal tympanic plate (Fig. 4). The anterior border of the osseous lamina has a broad notch delimited dorsally, near the capitular spine, by a small process. In the ventral aspect of this notch is a tiny groove, which based on the morphology in *Felis catus* [41] transports the chorda tympani nerve from the medial surface of the osseous lamina to the lateral surface of the tympanic plate.

**Figure 6:**
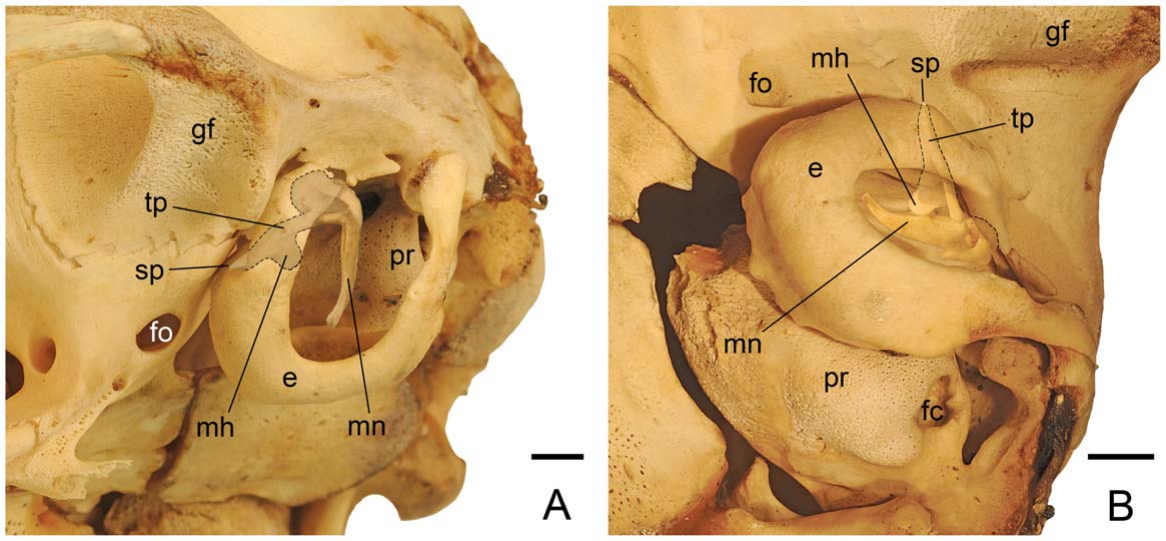
*Panthera pardus*, CM 21066, isolated malleus (reversed from right specimen) repositioned into the left middle ear. **A,** oblique lateral; **B,** ventral views. Scale bars = 3 mm. Abbreviations: **e,** ectotympanic; **fc,** fenestra cochleae (round window); **fo,** foramen ovale; **gf,** glenoid fossa; **mh,** mallear hook; **mn,** manubrium; **pr,** promontorium of petrosal; **sp,** spine of tympanic plate; **tp,** tympanic plate of rostral process.

In contrast to prior reports for felids in the anatomical literature, the rostral process of the leopard has a tympanic plate that is elongate, even longer than the manubrium (Figs. 4, 5). It has three sides tapering to a point distally; these are roughly dorsal, ventral, and lateral. The dorsal and ventral sides are formed by a high ridge of bone that lies at a 90° angle to the plane of the osseous lamina/PPA (Figs. 4A, C, 5B, D). This ridge is most pronounced proximally where it produces a distinct, curved process with an oval articular surface on its anterodorsal face. Wible [26] reported a similar process for the lipotyphlan *Solenodon paradoxus*, which contacted the tegmen tympani of the petrosal, and named it the petrosal process of the rostral process. In the leopard, this process articulates with a facet on the tegmen tympani and alisphenoid. Consequently, we chose a more generic name for this structure, the mallear hook, in order to be applicable to the condition in both the solenodon and the leopard. The ventral side of the leopard tympanic plate occupies the sulcus malleolaris on the anterior crus of the ectotympanic (Fig. 6). It has a faint groove running from the base of the mallear hook nearly to the distal tip (Fig. 5B, D); based on *Felis catus* [41], this groove likely transmits the rostral (anterior) tympanic artery, which supplies the tensor tympani muscle. The PPA extends onto the ventral side to the base of the mallear hook (Fig. 4A, C). The lateral side of the tympanic plate (Fig. 4B, D) includes the groove for the chorda tympani nerve noted above. Parallel to this groove and extending the length of the tympanic plate is a deep, open seam, interpreted as a remnant of the housing for Meckel’s cartilage (Fig. 4B, D). The leopard osseous lamina and tympanic plate do not have outer and inner lamellae fully comparable to the condition in the opossum (Fig. 1A, B). We interpret these in the leopard as the two sides of the groove for the chorda tympani (Figs. 4B, D, 5B, D), with the open seam on the lateral surface of the tympanic plate representing the interlamellar sulcus (Fig. 4B, D). The inner lamella is the base for the mallear hook.

The anterior and posterior views of the malleus (Fig. 5) show the tympanic plate of the rostral process and the manubrium do not occupy the same plane but diverge significantly.

In Figure 6, the isolated malleus is repositioned into the middle ear in oblique lateral and ventral views, and the part of the bone hidden by the anterior crus of the ectotympanic is shown with dashed lines. The ventral side of the tympanic plate fits into the sulcus malleolaris of the anterior crus. Only the distal tip of the tympanic plate, called here the spine, is visible medial to the glenoid fossa and the postglenoid process of the squamosal. On either side of the spine, the anterior crus contacts the skull base: medially, the alisphenoid, and laterally, the squamosal. The gap between the spine of the tympanic plate, anterior crus, and squamosal is the Glaserian fissure, which transmits the chorda tympani. The ectotympanic is U-shaped with a process of the squamosal nearly filling the tympanic incisure. The ectotympanic is not a simple ring, but is expanded into the anterior and medial walls of the middle ear. The dorsal part of this expansion in the anterolateral wall is visible as a narrow, horizontal shelf (Fig. 6B). In the posterolateral margin of this shelf is the ectotympanic notch on the dorsal surface of which is the sulcus malleolaris. The part of the tympanic plate posterior to the notch is called here the base. The prolongation of the PPA on the base fits into this notch. Extending medially from the base is the mallear hook, which articulates with the tegmen tympani and alisphenoid.

The morphology in the juvenile leopard resembles that of the cat as described by Jayne [16] except that (1) the process comparable to the ectotympanic hook of the cat (Fig. 3A) is part of the tympanic plate of the rostral process of the malleus in the leopard (Figs. 4, 5); (2) the short rostral process of the cat ends at the pit (ectotympanic notch) in the inner surface of the ectotympanic (Fig. 3A) to which it is attached by ligament, but in the leopard the elongate tympanic plate of the rostral process continues forward in the sulcus malleolaris to appear on the anterior face of the auditory bulla as the spine (Fig. 6); and (3) the PPA in the leopard has a central buttress (Fig. 4A, C).

**Figure 7:**
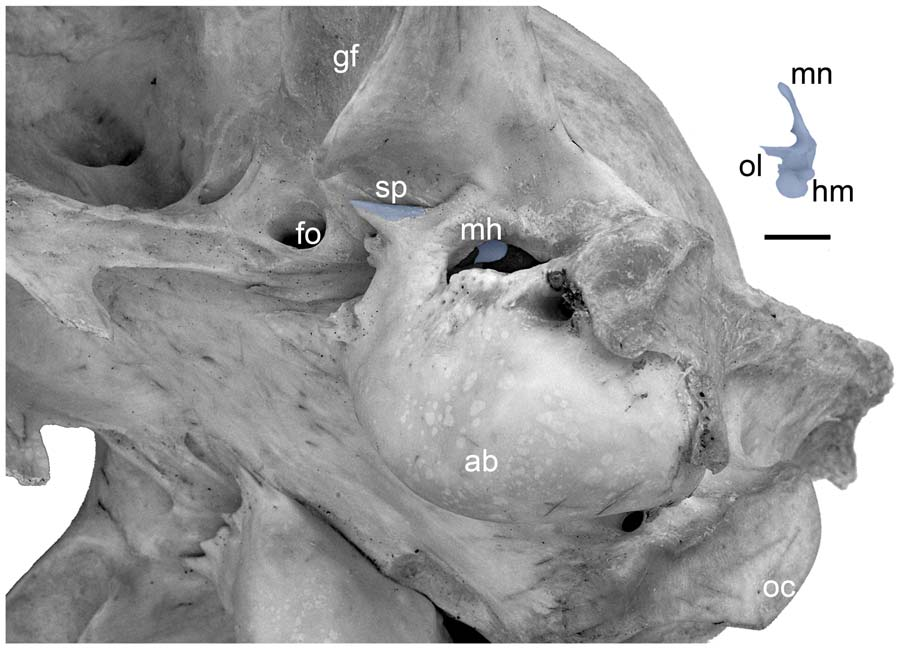
*Panthera pardus*, CM 57915, oblique lateral view of left basicranium with isolated partial malleus (blue) broken across the osseous lamina/PPA in medial view. The tympanic plate (blue) is preserved in situ; the spine is set off from the auditory bulla by sutures, but the mallear hook is fused to the bulla. Scale = 3 mm. Abbreviations: **ab,** auditory bulla; fo, foramen ovale; **gf,** glenoid fossa; **hm,** head of malleus; **mh,** mallear hook; **mn,** manubrium; **oc,** occipital condyle; **ol,** osseous lamina; **sp,** spine of tympanic plate.

CM 57915, adult female leopard, with a broken piece of each malleus found in the bottom of the specimen box (Fig. 7). The malleus is broken in two pieces. The isolated piece includes the head, manubrium, osseous lamina, and PPA; the last two parts have broken anterior edges that differ slightly between the right and left elements. These broken edges presumably would have connected to the other piece, the tympanic plate, which is attached to the auditory bulla. The spine of the tympanic plate is set off by sutures from the front of the bulla, but the mallear hook, which is visible through the external acoustic meatus, is fused to the inner wall of the bulla. The spine lies between narrow openings into the bulla; based on the cat, the medial opening is for the rostral tympanic artery and the lateral is the Glaserian fissure for the chorda tympani nerve (see fig. 2 in [41]).The isolated mallei have no surfaces with unfinished spongy bone. The muscular process is longer and the distal end of the manubrium is slightly spatulate compared to the juvenile, both of which are likely ontogenetic changes. We interpret two other differences as resulting from sexual dimorphism; the central buttress and PPA are not as robust in the adult female compared to the juvenile male. Although the greatest skull length of the juvenile is 66% that of the adult, the length of the malleus from head to manubrium in the juvenile is 20% greater than the adult.

CM 6422, adult leopard, with occiput, posterior basicranium, and ossicles missing except the tympanic plate of the malleus. This specimen is broken such that the inner (tympanic) aspect of the anterior wall of the bulla is visible. Lateral to the mallear hook is a paper thin, vertical lamina of bone, which we interpret as the broken connection between the osseous lamina/PPA and the base of the tympanic plate. On the anterior wall of the bulla, the spine of the tympanic plate is only partially set off by sutures.

#### *Felis catus*

CM 21412, female kitten with eyes still unopened upon death; no entotympanics are preserved; the malleus and ectotympanic are preserved in situ on the right side. This kitten repeats the juvenile leopard pattern with an elongate tympanic plate in the sulcus malleolaris ending in a spine and a prominent mallear hook on the base. However, the osseous lamina has a narrow, oval-shaped opening through it, close to its anterodorsal border, which we interpret as transmitting the chorda tympani nerve (see below). The ectotympanic is a simple ring except for the anterior crus, which is expanded to notch the rostral process, but lacks any indication of an ectotympanic hook.

CM 1407, juvenile cat with a full complement of deciduous teeth and no permanent dentition erupted; the ectotympanics and entotympanics are expanded to complete the osseous auditory bullae; the right bulla is opened but the ossicles are missing, except the tympanic plate of the mallear rostral process. The tympanic plate is preserved within the sulcus malleolaris, largely hidden by the underlying ectotympanic. The existence of the tympanic plate distinct from the ectotympanic is in evidence in two places. First, on the anterior face of the bulla, the tongue-shaped spine is set off by sutures. It separates openings for the rostral tympanic artery medially and the chorda tympani laterally (see fig. 2 in [41]). Second, within the middle ear, posterior to the ectotympanic notch is a suture between the lateral border of the base of the tympanic plate and the ectotympanic. A broken seam along the ventral edge of the base indicates where the remainder of the malleus (osseous lamina, head, and manubrium) was broken off. In contrast to the younger kitten, the anterior border of the mallear hook is fused to the ectotympanic, by which it now resembles an ectotympanic hook.

CM 28447, adult male cat with left auditory bulla opened; the left ossicles are missing, except the tympanic plate of the mallear rostral process. This specimen repeats the pattern of the juvenile cat, except that the spine of the tympanic plate is now fully fused to the ectotympanic on the anterior face of the bulla. On the specimen’s right side, the intact malleus with the osseous lamina and a prominent PPA continuous with the tympanic plate at the ectotympanic notch is visible through the external acoustic meatus. The presence of an opening in the osseous lamina as reported above for the newborn cat cannot be confirmed as this part of the malleus is not visible.

**Figure 8:**
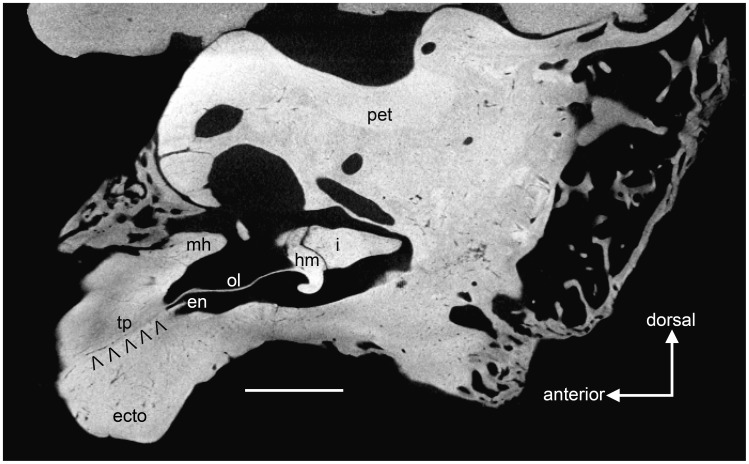
*Felis catus*, adult, longitudinal section through the middle ear generated by Avizo®7 from CT data [42] (see text for methods). Seam between tympanic plate of mallear rostral process and ectotympanic is marked by carets. Scale = 2 mm. Abbreviations: **ecto,** ectotympanic; **en,** ectotympanic notch; **hm,** head of malleus; **i,** incus; **mh,** mallear hook; **ol,** osseous lamina; **pet,** petrosal; **tp,** tympanic plate of rostral process.

**Figure 9:**
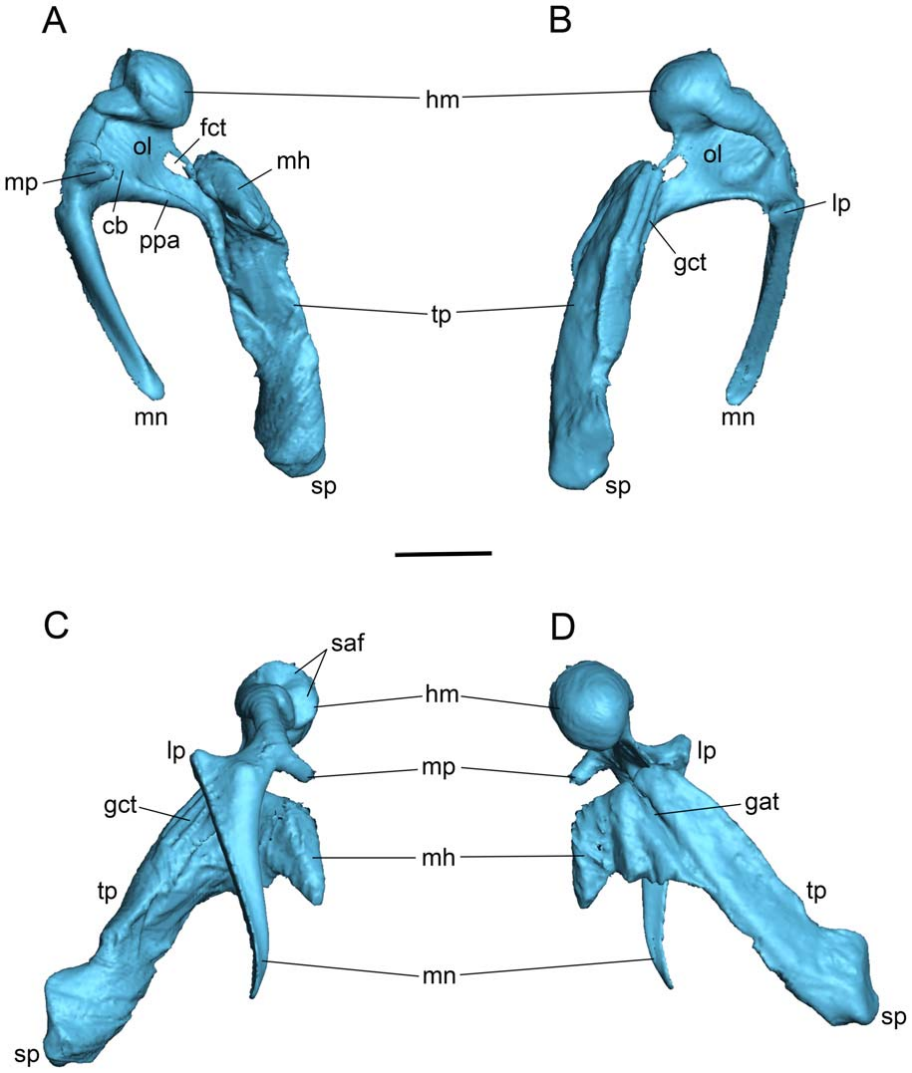
*Felis catus*, model of adult left malleus (reversed from right specimen) based on CT data [42]. **S**ee text for methods; the margins of the tympanic plate were distinguishable from the auditory bulla in most, but not all slices. **A,** medial; **B,** lateral; **C,** anterior; **D,** posterior views. Scale = 2 mm. Abbreviations: **cb,** central buttress; **fct,** foramen for chorda tympani; **gat,** groove for anterior tympanic artery; **gct,** groove for chorda tympani; **hm,** head of malleus; **lp,** lateral process; **mh,** mallear hook; **mn,** manubrium; **mp,** muscular process; **ol,** osseous lamina; **ppa,** pars processus anterioris; **saf,** superior articular facet; **sp,** spine of tympanic plate; **tp,** tympanic plate of rostral process.

Adult cat (CT data from [42]), Salih et al. [42] have recently published an open access model of the auditory ossicles based on CT scans of an adult cat. Their malleus (see fig. 1A in [42]) resembles the element illustrated by Jayne [16] (Fig. 1C, D). Salih et al. [42] have provided us with their original CT data, which was scanned at a resolution of 23.7 µm. We imported their 499 roughly transverse slices of a right petrosal, auditory bulla, and ossicles into Avizo®7 (VSG, Burlington, Massachusetts). These slices were assembled and resampled into two additional planes, roughly horizontal and longitudinal. Study of the sets of slices revealed that the osseous lamina/PPA is continuous with the inner surface of the anterior wall of the bulla (Fig. 8), as noted by some previous authors [23]. Additionally, we found that the part of the bulla to which the osseous lamina/PPA connected is distinguished from the main bulla by seams and density differences (Fig. 8) in many, though not all slices. We followed these features through the three planes of section, produced a model of the malleus with what we identify as the tympanic plate (Fig. 9), and include a movie of the model in Movie S1. The mallear model is remarkably similar to the element of the juvenile leopard (Figs. 4, 5). Here, we describe the major differences between the two.

On the head, the superior articular facet is more distinctly separated by a central ridge into subequal medial and lateral surfaces (Fig. 9C) and a capitular spine is not evident (Fig. 9A, B, D). On the osseous lamina, the notch in the anterior margin in the leopard through which the chorda tympani runs (Fig. 4) is enclosed by a narrow bar of bone to form a oval opening in the cat (Fig. 9A, B). A central buttress is present (Fig. 9A), but weaker than in the leopard (Fig. 4A, C). The entire tympanic plate is more robust, with the spine having a broad distal end and the mallear hook more pronounced and pointed (Fig. 9C, D). The grooves for the chorda tympani nerve and rostral tympanic artery are also more pronounced; the former is on the lateral surface of the tympanic plate leading from the foramen in the osseous lamina (Fig. 9B), whereas the latter is on the dorsal surface along the base of the mallear hook (Fig. 9D).

Figure 3B shows our interpretation of the adult cat anatomy based on these specimens. The tympanic plate of the rostral process is fused to the anterior crus of the ectotympanic; the ectotympanic hook of Jayne [16] is the mallear hook of the rostral process. Contra Jayne [16], the rostral process does not end at the ectotympanic notch (as in Fig. 3A) but continues anteriorly as the tympanic plate.

### Nandiniidae

#### *Nandinia binotata*

**Figure 10:**
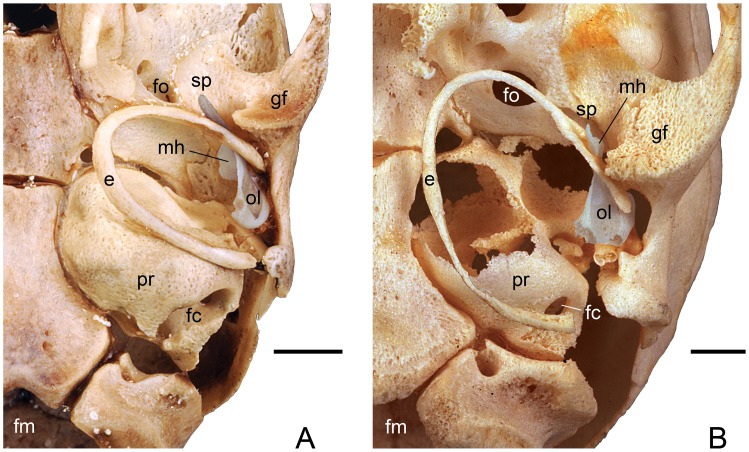
Left auditory regions in ventral view. **A,** *Nandinia binotata*, AMNH 207730, newborn; **B,** *Canis latrans*, AMNH 181990, two- to three-day old pup. The malleus is shaded in blue; the manubrium has not yet ossified in either; the promontorium of the petrosal is not fully ossified in B. Scale bars = 2 mm. Abbreviations: **e,** ectotympanic; **fc,** fenestra cochleae (round window); **fm,** foramen magnum; **fo,** foramen ovale, **gf,** glenoid fossa; **mh,** mallear hook; **ol,** osseous lamina; **pr,** promontorium of petrosal; **sp,** spine of tympanic plate.

AMNH 207730, newborn African palm civet with no erupted teeth; no entotympanics are preserved; the mallei and ectotympanics are preserved in situ (Fig. 10A). This specimen repeats the pattern of the kitten, CM 21412. The elongate tympanic plate of the rostral process occupies the sulcus malleolaris on the ectotympanic, ends in a spine, and has a mallear hook (Fig. 10A). The main differences are the absence of a foramen in the osseous lamina and that the ectotympanic is a simple ring without a notch for the tympanic plate.

AMNH 51471, juvenile male African palm civet with deciduous dentition; entotympanics are preserved; the mallei and ectotympanics are preserved in situ. Through the external acoustic meatus, the base of the tympanic plate is visible fitting into the ectotympanic notch. The mallear hook and spine are fused to the ectotympanic, but the spine can be distinguished by its bony composition; the surrounding ectotympanic is riddled with tiny holes, whereas the spine is not. The PPA extends along the proximal two-thirds of the osseous lamina.

AMNH 51513, adult female African palm civet with ossicles preserved in situ on left side. This adult repeats the pattern of the juvenile, with the osseous lamina continuous with the tympanic plate at the ectotympanic notch.

### Viverridae

#### *Genetta maculata*

**Figure 11:**
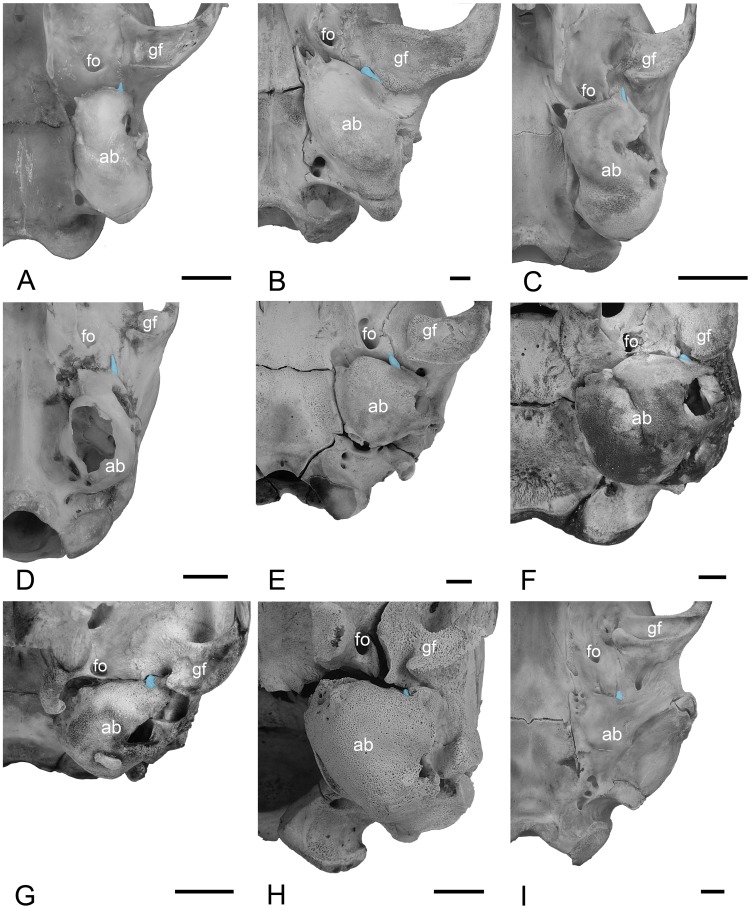
Left basicrania in ventral or oblique ventral view with the spine of the tympanic plate of the mallear rostral process highlighted in blue. **A,** *Genetta maculata*, CM 85593 (reversed from right side); **B,** *Crocuta crocuta*, CM 2981 (reversed from right side); **C,** *Herpestes javanicus*, CM 45482; **D,** *Eupleres goudotii*, AMNH 100462 (reversed from right side; auditory bulla broken); **E,** *Ursus americanus*, CM teaching collection (young of CM 63096); **F,** *Erignathus barbatus*, CM 6545; **G,** *Procyon lotor*, CM 51852; H, *Mellivora capensis*, CM 10327: basioccipital is missing; **I,** *Lontra canadensis*, CM 18863 (reversed from right side). Scale bars = 5 mm. Abbreviations: **ab,** auditory bulla; **fo,** foramen ovale; **gf,** glenoid fossa.

CM 85593, adult female rusty-spotted genet with ossicles in situ. The spine of the tympanic plate is set off by sutures from the underlying ectotympanic (Fig. 11A). The spine comes to a point that is appressed against the alisphenoid a short way in front of the bulla. It separates openings for the rostral tympanic artery medially and chorda tympani laterally. Through the external acoustic meatus, the PPA and osseous lamina are seen to be continuous with the base of the tympanic plate and its mallear hook. The osseous lamina does not have a foramen or notch in its anterior margin.

### Hyaenidae

#### *Crocuta crocuta*

CM 2981, juvenile male spotted hyaena with deciduous teeth and erupting permanent carnassials; the auditory bullae are fully formed and obscure the middle ear. Bilaterally, the spine of the tympanic plate is set off by sutures from the ectotympanic on the anterior face of the bulla (Fig. 11B). In contrast to the finger-like distal tympanic plate of the other carnivorans, here the structure is flattened onto the overlying squamosal and mediolaterally expanded. It forms the lateral wall of the Glaserian fissure.

### Herpestidae

#### *Herpestes javanicus*

CM 45482, juvenile female small Asian mongoose with deciduous dentition; the complete auditory bullae obscure the middle ear. The spine of the tympanic plate is set off by sutures from the ectotympanic on the anterior face of the bulla (Fig. 11C). It comes to a point that barely extends beyond the bulla and forms the lateral wall of the Glaserian fissure.

### Eupleridae

#### *Eupleres goudotii*

AMNH 100462, adult female falanouc with mallei in situ, but hidden behind the tympanic membrane. The spine of the tympanic plate is set off by sutures from the ectotympanic on the anterior face of the bulla (Fig. 11D). It is an elongate, finger-like process that extends anteriorly away from the bulla. It forms the floor of the Glaserian fissure.

#### *Fossa fossana*

AMNH 188208, adult male Malagasy civet or fanaloka with mallei in situ, but continuity with the rostral process cannot be confirmed through the external acoustic meatus. The spine of the tympanic plate is set off by sutures from the ectotympanic on the anterior face of the bulla. It is robust where it juts out from the ectotympanic and tapers to a point. It forms the floor for the Glaserian fissure and does not contact the skull base. The PPA is prominent in only the proximal half of the osseous lamina. The latter lacks a foramen or notch in its anterior margin.

### Canidae

#### *Canis latrans*

AMNH 181990, two- to three-day old coyote pup; no entotympanics are preserved; mallei and ectotympanics are preserved in situ (Fig. 10B). This specimen resembles the newborn feliforms described above, *Felis* and *Nandinia* (Fig. 10A), with a long tympanic plate in the sulcus malleolaris of the ectotympanic. The tympanic plate has a pointed spine extending beyond the anterior crus. Its lateral surface, which forms the medial wall of the Glaserian fissure, has a faint sulcus for the chorda tympani. There is a triangular mallear hook that is smaller than that in the two feliforms. The ectotympanic is a simple ring with a notch for the base of the tympanic plate. As in the *Nandinia* newborn, the manubrium is not yet ossified, a foramen in the osseous lamina is lacking, and a weak PPA is present in the coyote.

CM 13302, juvenile male coyote with full deciduous dentition; the auditory bullae are complete; the mallei are not preserved except for the tympanic plate. The spine of the tympanic plate is partially fused to the ectotympanic on the anterior face of the bulla; only its ventral side is distinct. It comes to a point, but does not project beyond the ectotympanic. Its lateral surface has a sulcus for the chorda tympani. Medial to the spine is a small foramen presumably for the rostral tympanic artery [17]. The medial aspect of the mallear hook is visible through the external acoustic meatus, but additional details are hidden.

#### *Canis lupus familiaris*

CM 23065, newborn male dog skull in pieces. The right malleus and ectotympanic are isolated and resemble the patterns in the newborn feliforms and coyote (Fig. 10). The malleus has a long tympanic plate with a spine extending beyond the anterior crus of the ectotympanic. It has a tiny mallear hook at the base. The ring-shaped ectotympanic has a notch and sulcus malleolaris. The osseous lamina is without a foramen; the PPA is lacking, but is well developed without a central buttress in the adult (see fig. 20–11A in [17]).

### Ursidae

#### *Ursus americanus*

CM teaching collection [young of CM 63096], American black bear cub with deciduous dentition; the auditory bullae are complete; the mallei are preserved in situ. A well-developed spine of the tympanic plate is set off by sutures from and extends well rostral to the anterior face of the auditory bulla (Fig. 11E). The Glaserian fissure lies lateral to the spine. Through the external acoustic meatus, the malleus posterior to the tympanic plate is visible. Continuity between the spine in front and the osseous lamina/PPA behind cannot be confirmed, because they are separated by the anterior crus of the ectotympanic. However, in light of the short distance between them, continuity seems highly likely. The osseous lamina is without a foramen or notch in its anterior margin.

CM 13295, male American black bear cub with deciduous dentition and M1 (first molar) erupting; the auditory bullae are complete. We removed a wedge of the left auditory bulla with a small, handheld circular saw. The malleus was initially preserved in situ with the tympanic plate disappearing into the ectotympanic notch and the mallear hook fused to the ectotympanic. However, the vibrations of the saw caused the malleus to break between the base of the tympanic plate posterior to the ectotympanic notch and the osseous lamina/PPA, with the line of breakage still visible. There is no evidence of a separate spine on the anterior face of the bulla.

### Phocidae

#### *Erignathus barbatus*

CM 6545, juvenile male bearded seal with complete deciduous dentition; the auditory bullae are complete but sutures between the ecto- and entotympanics are preserved. Doran’s illustration and description of the malleus for this form (see plate LIX, fig. 29 in [6]) do not indicate the presence or size of the osseous lamina. The isolated right malleus of CM 6545 shows that the osseous lamina is broken very close to the head and neck. There is no indication of the PPA on the narrow bit of osseous lamina that is preserved. On the skull, a spine of the tympanic plate is found on the anterior wall of the bulla (Fig. 11F), although partially fused to the ectotympanic. It is a short rod that tapers distally and forms the medial wall of the Glaserian fissure.

### Odobenidae

#### *Odobenus rosmarus*

CM 15333, subadult male walrus with permanent dentition erupting; the skull is in pieces with the auditory bullae fully formed. Two isolated mallei are preserved that generally resemble those figured by Doran (see plate LIX, figs. 21, 22 in [6]) and Wyss (see fig. 3B in [12]) with a rudimentary osseous lamina and no indication of the PPA. However, it is clear from the CM specimens that the osseous lamina is broken, although it is not known how much is missing. The auditory bullae show no sign of a separate tympanic plate spine. A sulcus for the chorda tympani at the Glaserian fissure indicates where the spine would be expected.

### Otariidae

#### *Callorhinus ursinus*

CM 135, juvenile Northern fur seal with permanent dentition erupting; the auditory bullae are complete and the sutures between the ecto- and entotympanics are partially preserved. There is no indication of the tympanic plate spine on the anterior face of the bulla. An isolated left malleus preserves a triangular osseous lamina with a PPA on the ventral margin. The ventral third of the anterior face of the lamina is broken. Wyss photographed a malleus for this taxon (see fig. 3C in [12]) with an even smaller osseous lamina than observed here, presumably resulting from more damage.

### Ailuridae

#### *Ailurus fulgens*

CM 1689 and 17508, adult red pandas. The only two red pandas available for study have no evidence for a separate tympanic plate spine on the anterior face of the bulla. The mallei are preserved in situ in CM 17508 but cannot be fully examined through the external acoustic meatus.

### Mephitidae

#### *Mephitis mephitis*

CM 5527, subadult male striped skunk with complete auditory bullae, obscuring the middle ear. The tympanic plate spine is distinct on the anterior face of the bulla. The spine has a short point that is appressed against the squamosal on the skull base and is underlain by the ectotympanic. The Glaserian fissure lies between these three elements. The left spine is one-third the width of the right.

### Procyonidae

#### *Procyon lotor*

CM 51852, juvenile female raccoon with erupting deciduous dentition; the auditory bullae are complete and the sutures between the ecto- and entotympanics are fusing; the mallei are preserved in situ. The tongue-shaped spine of the tympanic plate is set off by sutures on the anterior face of the bulla (Fig. 11G). It projects only slightly beyond the ectotympanic and forms the floor for the Glaserian fissure. Within the middle ear, the osseous lamina disappears at the ectotympanic notch and the mallear hook is in sutural contact anteriorly with the anterior crus. The PPA is present but only extends along the proximal two-thirds of the osseous lamina. A foramen or notch in the anterior margin of the osseous lamina is absent.

CM 92638, juvenile male raccoon with erupted deciduous dentition; the seam between the ecto- and entotympanic in the auditory bulla is fused; the mallei are preserved in situ. Although only slightly older than the prior specimen, the spine of the tympanic plate is nearly completely fused to the neighboring ectotympanic; only the tip remains separate. It does not project beyond the ectotympanic, but is recessed in a depression. The Glaserian fissure lies between the tip of the spine and ectotympanic. Features of the tympanic plate base and mallear hook are not visible through the external acoustic meatus.

### Mustelidae

#### *Mellivora capensis*

CM 10327, juvenile honey badger with deciduous dentition erupting; the auditory bullae are complete; the mallei are missing except the tympanic plate. The tongue-shaped spine of the tympanic plate is nearly surrounded by the ectotympanic on the anterior face of the bulla (Fig. 11H). It does not project beyond the ectotympanic but is housed in a depression. The Glaserian fissure is positioned posterolateral to the spine, between the ectotympanic and squamosal. Visible through the external acoustic meatus posterior to the ectotympanic notch are the base of the tympanic plate, the mallear hook, and a small piece of the osseous lamina.

CM 57985, adult male honey badger. The spine of the tympanic plate and the depression in the ectotympanic that it occupied in the juvenile are no longer evident on the anterior face of the bulla. The spine has either fused to or has been overgrown by the ectotympanic. Details within the middle ear are obscured by the bulla.

#### *Martes pennanti*

CM 40440, adult fisher skull in pieces; mallei are not preserved except for the tympanic plate. The roof of the middle ear anterior to the right petrosal is exposed because the auditory bulla on that side is isolated. The bulla was broken in such a way that the tympanic plate split, with the dorsal half preserved in situ on the skull base and the ventral half in the sulcus malleolaris of the ectotympanic. The tympanic plate is distinguished from its neighbors by seams and a whiter color. It is a rod that tapers to a point, but that point is covered by the repositioned bulla and not visible on the skull base, as a similar spine on the ectotympanic covers it. A mallear hook is present but its full extent is uncertain due to damage.

#### *Lontra canadensis*

CM 18863, adult female North American river otter skull with the left ear region isolated and opened. The left middle ear was opened such that the medial aspect of the malleus is visible. Both the base and spine of the tympanic plate are visible, but the ectotympanic obscures the portion in between. The base is continuous with the prominent PPA and osseous lamina. The mallear hook is fused to the ectotympanic. A crack across the osseous lamina near the base likely indicates where the malleus usually breaks in other studied carnivorans. On the specimen’s right side, the triangular spine of the tympanic plate is appressed against the skull base near the alisphenoid-squamosal suture (Fig. 11I). It is separated by sutures from the alisphenoid medially and the ectotympanic posteriorly, but fused to the squamosal laterally. Its medial surface is grooved by the chorda tympani and the Glaserian fissure is at the juncture of the spine, alisphenoid, and ectotympanic.

#### *Mustela erminea*

CM 7538 and 17834, adult stoats with isolated mallei. Fleischer [21] reported a short rostral process for *M. erminea* with the triangular osseous lamina not coupled to the ectotympanic. We were not able to confirm or deny this with our available specimens. The isolated CM mallei of *M. erminea* resemble the element illustrated by Fleischer (see fig. 53 in [21]), but clearly show that the osseous lamina and prominent PPA are broken.

## Discussion and Conclusions

Our study provides evidence that a long tympanic plate of the rostral process is anchored to the ectotympanic in a diverse assortment of juvenile and adult carnivorans, and, with age, the fate of the tympanic plate is sutural fusion to the auditory bulla. A tympanic plate distinct from the bulla was found in representatives of 12 of the 15 extant families (Fig. 11), including all feliform families and the basal caniform families [43], suggesting this morphology is primitive for Carnivora. The existence of a tympanic plate could not be denied or confirmed in the available sample for three families, Odobenidae, Otariidae, and Ailuridae. A consequence of our observation concerns the composition of the carnivoran auditory bulla. It has long been recognized that the carnivoran bulla is a composite of the ectotympanic and at least two entotympanics [44], [45], [46]. The sutures between these elements are fully fused in adults, except in the nandiniid *Nandinia binotata* in which the main entotympanic remains cartilaginous throughout ontogeny [45]. The tympanic plate of the rostral process must be included on this list of bullar elements for the taxa expressing it in our study. The fusion of the tympanic plate to the bulla postdates the fusion of the ectotympanic and entotympanics in our sample with the exception of *N. binotata*.

We are not the first to make observations of a long tympanic plate in carnivorans. Fleischer [21] reported one coupled to the ectotympanic for the canids *Canis lupus* and *Vulpes*, the otariid *Arctocephalus pusillus*, the phocid *Phoca vitulina*, and the mustelid *Martes*. However, Fleischer’s observations have not been noted by subsequent authors, possibly because they were written in German.

Another consequence of our findings concerns the carnivoran gonial. In the prior anatomical literature, the absence of a tympanic plate to the carnivoran malleus (Fig. 1C, D) implies that its embryonic precursor, the gonial, either resorbs in the adult or is rudimentary to begin with. In contrast, our findings of a well-developed tympanic plate reveal that the gonial must be a substantial element embryonically. This is in accord with embryonic results for the dog [17], [39].

Our study provides further evidence that in a subset of our carnivoran sample the tympanic plate is continuous with the rest of the malleus through the osseous lamina/PPA, as has been reported in all other mammals with a tympanic plate studied to date. To verify this in more taxa would require either destructive procedures to open more bullae or the acquisition of additional CT imagery, both of which are beyond the scope of the current report. Here, we found continuity in adults, juveniles, and newborns of the felid *Felis catus* (Figs. 8, 9) and the nandiniid *Nandinia binotata* (Fig. 10A); in adults of the viverrid *Genetta maculata* and the mustelid *Lontra canadensis*; in juveniles of the felid *Panthera pardus* (Figs. 4–6), the ursid *Ursus americanus*, and the procyonid *Procyon lotor*; and in newborns of the canids *Canis latrans* (Fig. 10B) and *Canis lupus familiaris*. In addition, Fleischer [21] reported continuity between the osseous lamina and tympanic plate in adults of the canid *Vulpes*, the otariid *Arctocephalus pusillus*, the phocid *Phoca vitulina*, and the mustelid *Martes*. In total, adult representatives of the basal feliform and caniform families [43] exhibit continuity, suggesting this is the primitive morphology for Carnivora.

The notion that the carnivoran malleus has a short rostral process that is not coupled to the ectotympanic (i.e., without a tympanic plate; Figs. 1C, D, 3A) is rampant in the comparative anatomical and systematics literature (e.g., [12], [16], [17], [47], [48]). This is in part because many studies utilize major anatomical reference works on the domestic dog and cat as exemplars, and these have depicted the malleus ending rostrally with the osseous lamina and PPA [14], [15], [16], [17]. Additionally, this mallear shape has been modeled for the cat based on CT data in recent physiological acoustics reports [42], [49]. Prior anatomical studies of mallei from nearly 100 additional carnivoran taxa including representatives from all 15 extant families essentially have repeated this view [6], [10], [11], [12], [13], [19], [20].

What accounts for the discrepancy between these reports and that presented here and in Fleischer [21] (cf. Fig. 3A, B)? Individual variation is an unlikely explanation. Shape differences in the stapes have been noted previously in various taxa [50], [51], but the discrepancy in the presence/absence of the mallear tympanic plate of carnivoran genera is dramatically more extreme. Ontogenetic differences are another possible explanation for some discrepancies. It is true that we were able to confirm continuity between the osseous lamina/PPA and the tympanic plate in adults for only a subset of our carnivoran sample. For other adults, we cannot rule out the possibility that the continuous osseous lamina and tympanic plate of early stages (such as in the *Ursus americanus* cubs) are decoupled in the adult stage, leaving only connective tissue between them. However, because a tympanic plate disassociated from the rest of the malleus has not yet been described for any adult carnivoran, or any adult mammal for that matter, this is not a robust explanation.

We believe that the principal reasons for the discrepancy in reports on the carnivoran malleus are the search image provided by the prior authoritative literature and the preparations available for study. In essence, the search image based on the anatomical literature is for a short rostral process without a tympanic plate. If a carnivoran malleus was found that matched this search image, there would be no reason to question its completeness, even if the thin osseous lamina showed evidence of damage.

For our study, we had a variety of preparations, including newborn, juvenile, and adult specimens with mallei in situ. In addition, we often found mallei in the bottom of museum specimen boxes, even in those housing skeletons in addition to skulls. Presumably, something in the preparation process dislodged these small elements from the middle ear. With the exception of the juvenile leopard, *Panthera pardus*, CM 21066, these isolated mallei were broken near the juncture of the tympanic plate with the osseous lamina/PPA, breakage facilitated by the delicate nature of this juncture and the tight attachment and frequent fusion of the tympanic plate to the ectotympanic. We also unintentionally broke a malleus of *Ursus americanus* in precisely this location by merely cutting a window in the bulla; the vibrations from the circular say were sufficient to damage and dislodge this delicate element. The morphology resulting from the breakage of the bear and other detached mallei matches that previously reported as complete mallei in the vast majority of the carnivoran literature. It was the fortuitous discovery of the complete juvenile leopard malleus that provided us with a different search image; an ossicle that includes the tympanic plate base at the ectotympanic notch and the spine on the anterior face of the bulla (Fig. 3B). Access to juvenile specimens was another critical aspect of this study, but not essential, because we still encountered five adult carnivorans from three families with the spine set off from the ectotympanic by sutures (i.e., the felid *Panthera pardus*, the viverrid *Genetta maculata*; the euplerids *Eupleres goudotii* and *Fossa fossana*; and the mustelid *Lontra canadensis*) (Figs. 7, 11A, E, I).

We also discovered characteristics of the malleus that may be of systematic value within Carnivora, or even more broadly. One feature of the rostral process occurring in most of the studied carnivorans is the presence of the mallear hook, which articulates with the skull base, at a minimum with the tegmen tympani of the petrosal. The only carnivoran that this structure has been noted in previously is *Felis catus* where Jayne [16] identified it as a hook belonging to the ectotympanic (Fig. 3A). This is presumably because Jayne [16] only studied adults where the mallear hook is fused to the anterior crus. We identified a mallear hook in the felids *Panthera pardus* (Figs. 4, 5, 7) and *Felis catus* (Fig. 9); the nandiniid *Nandinia binotata* (Fig. 10A); the viverrid *Genetta maculata*; the canids *Canis latrans* (Fig. 10B) and *Canis lupus familiaris*; the ursid *Ursus americanus*; the procyonid *Procyon lotor*; and the mustelids *Mellivora capensis*, *Martes pennanti*, and *Lontra canadensis*. The incidence of the mallear hook in the remaining specimens could not be confirmed or denied. In light of its broad distribution in feliforms and caniforms, a mallear hook is likely primitive for Carnivora. The only other mammal for which a mallear hook has been reported is the lipotyphlan *Solenodon paradoxus* [26].

Another unusual feature with a narrower distribution is the opening for the chorda tympani nerve in the anterior margin of the osseous lamina, which we found only in felids; this is a notch in the leopard *Panthera pardus* (Fig. 4) and an enclosed foramen in the cat *Felis catus* (Fig. 9A, B). Based on prior studies illustrating the osseous lamina in a wide range of felids [11], a notch is ubiquitous although not universally reported. Restudy of this may provide additional instances within felids where the notch is enclosed to a foramen by a delicate spicule of bone as in the cat (Fig. 9A, B).

Our discovery of this discrepancy in descriptions of the carnivoran rostral process raises questions about the validity of reports in the literature of reduced rostral processes in other mammalian groups. A perusal of the figures in Doran [6] reveals a number of placental clades depicted with short rostral processes lacking tympanic plates (e.g., artiodactyls, primates, pholidotans). We are not suggesting that all instances of short rostral processes in the literature have suffered the same problem as reported here for Carnivora, rather that these morphologies are deserving of another look.

**Figure 12:**
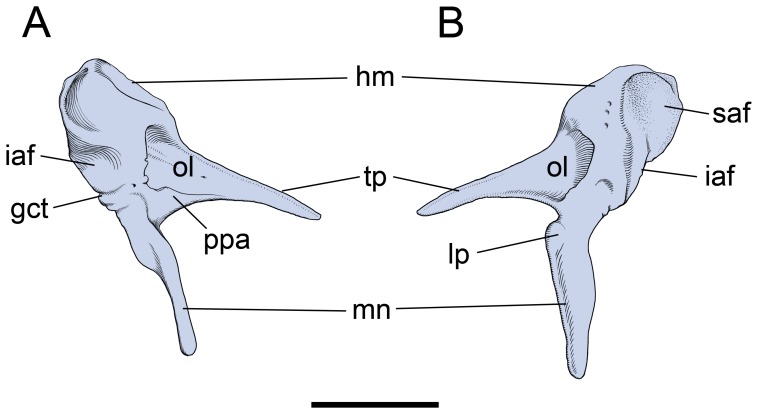
*Manis pentadactyla*, AMNH 60019, left malleus (reversed from right specimen). A, medial; **B,** lateral views. Scale bar = 2 mm. Abbreviations: **gct,** groove for chorda tympani; **hm,** head of malleus; **iaf,** inferior articular facet; **lp,** lateral process; **mn,** manubrium; **ol,** osseous lamina; **ppa,** pars processus anterioris; **saf,** superior articular facet; **tp,** tympanic plate of rostral process.

In that regard, we chose to reexamine pangolins (Pholidota), the extant sister group of Carnivora (e.g., [52]). This order includes only eight extant species in three genera [53] and is reported to have short rostral processes [6], [21], [53], [54] resembling that illustrated by Jayne [16] for the domestic cat (Fig. 1C, D). We found mallei of an adult Chinese pangolin, *Manis pentadactyla*, AMNH 60019, to have a long, thin tympanic plate that is slightly shorter than the manubrium (Fig. 12). The tympanic plate occupies the sulcus malleolaris on the ectotympanic, but does not have a visible spine, as the plate does not extend as far anteriorly as the ectotympanic. In addition, a juvenile tree pangolin, *Phataginus tricuspis*, CM 41123, with mallei and ectotympanic in situ has the same arrangement with a thin tympanic plate in the sulcus malleolaris and no spine on the anterior face of the bulla. Combined with our data here, a long rostral process with a tympanic plate coupled to the ectotympanic would characterize the clade of Carnivora and Pholidota. Neither of the two pangolin species examined had a mallear hook (Fig. 12).

The relative size of the rostral process has been used as a character in phylogenetic analyses within Carnivora [12], [43], [48], as well as in broader analyses of mammals and their extinct relatives [55]. Obviously, appropriate usage of this feature as a character is dependent on properly recognizing the homology across taxa, which requires precise anatomical identification of all component structures. A proper identification of the size of the rostral process is not only relevant to phylogenetic analysis; the malleus functions with the other ossicles to transmit sound waves from the tympanic membrane to the fluid-filled inner ear. Models of how the ossicular chain functions differ dependent on the degree of connection between the malleus and ectotympanic; these connections have been typically broken into anchored vs. freely mobile types, with a transitional type reflecting the continuum between them [56]. The opossum malleus is clearly the anchored (ancestral) type, with a thick connection between the osseous lamina, the tympanic plate, and ectotympanic (Fig. 1A, B), and the human malleus is unquestionably freely mobile, with only a ligamentous connection to the anterior tympanic wall [56]. While our model of the cat malleus (Fig. 9) more closely resembles that of the opossum, in having a fully ossified connection, it is not the anchored type. The felid connection between the osseous lamina and tympanic plate is not thick, but rather so tenuous that it easily breaks in museum preparations and is possibly flexible in life. A similar thin and flexible connection between the osseous lamina and tympanic plate has been described in the lipotyphlan mole *Talpa* [57], which is said to have a freely mobile type of malleus [56], [57]. Our study has clearly demonstrated that the carnivoran malleus repeats this pattern.

## Supporting Information

Movie S1 Movie of model of left malleus of adult cat, *Felis catus*. Model was generated in Avizo®7 from CT data provided by Sahil et al. [42].
(MOV)
